# *NRAS* and *EPHB6* mutation rates differ in metastatic melanomas of patients in the North Island versus South Island of New Zealand

**DOI:** 10.18632/oncotarget.9351

**Published:** 2016-05-13

**Authors:** Angela M. Jones, Peter Ferguson, Jacqui Gardner, Serena Rooker, Tim Sutton, Antonio Ahn, Aniruddha Chatterjee, Vivienne M. Bickley, Makhdoom Sarwar, Patrick Emanuel, Diane Kenwright, Peter R. Shepherd, Michael R. Eccles

**Affiliations:** ^1^ Capital and Coast District Health Board, Wellington, New Zealand; ^2^ Department of Pathology and Molecular Medicine, Wellington School of Medicine and Health Sciences, University of Otago, Wellington, New Zealand; ^3^ Anatomical and Molecular Pathology, Canterbury Health Laboratories, Christchurch, New Zealand; ^4^ Pathlab Bay of Plenty, Tauranga, New Zealand; ^5^ Department of Pathology, Dunedin School of Medicine, University of Otago, Dunedin, New Zealand; ^6^ Maurice Wilkins Centre for Molecular Biodiscovery, Auckland, New Zealand; ^7^ Department of Obstetrics and Gynaecology, Christchurch School of Medicine, University of Otago, Christchurch, New Zealand; ^8^ Anatomic Pathology Services, Auckland District Health Board, New Zealand; ^9^ Department of Pathology and Molecular medicine, University of Auckland, Auckland, New Zealand

**Keywords:** melanoma, mutations, NRAS, BRAF, EPHB6

## Abstract

Melanoma, the most aggressive skin cancer type, is responsible for 75% of skin cancer related deaths worldwide. Given that New Zealand (NZ) has the world's highest melanoma incidence, we sought to determine the frequency of mutations in NZ melanomas in recurrently mutated genes. NZ melanomas were from localities distributed between North (35°S-42°S) and South Islands (41°S-47°S). A total of 529 melanomas were analyzed for *BRAF* exon 15 mutations by Sanger sequencing, and also by Sequenom MelaCarta MassARRAY. While, a relatively low incidence of *BRAF^V600E^* mutations (23.4%) was observed overall in NZ melanomas, the incidence of *NRAS* mutations in South Island melanomas was high compared to North Island melanomas (38.3% vs. 21.9%, P=0.0005), and to The Cancer Genome Atlas database (TCGA) (38.3% vs. 22%, P=0.0004). In contrast, the incidence of *EPHB6^G404S^* mutations was 0% in South Island melanomas, and was 7.8% in North Island (P=0.0002). Overall, these data suggest that melanomas from geographically different regions in NZ have markedly different mutation frequencies, in particular in the *NRAS* and *EPHB6* genes, when compared to TCGA or other populations. These data have implications for the causation and treatment of malignant melanoma in NZ.

## INTRODUCTION

New Zealand (NZ) has the highest incidence rate of melanoma in the world [1], with new registrations occurring at a rate of 36.9 per 100,000 in the NZ population in 2012, when age-standardized to the WHO standard population (Wellington: Ministry of Health. www.health.govt.nz/publication/cancer-new-registrations-and-deaths-2012).

The cause of the high melanoma incidence in NZ is thought to be mainly due to the high levels of solar UV-radiation exposure, which the NZ population is exposed to, especially during the summer months. Solar UV radiation exposure is widely accepted as a key risk factor for cutaneous melanomas, although the latter may arise on both sun-exposed as well as non-sun-exposed skin sites [2]. Many melanomas develop in association with nevi on the skin, the number of which is proportional to childhood sun exposure, as well as to genetic factors [3]. In contrast, melanomas may also develop with no prior association with nevi. The sequencing of melanoma genomes has shown that melanomas frequently contain high mutation loads; on average melanomas contain the highest mutation load of all cancer types, including high levels of UV signature mutations, such as C>T nucleotide transitions [4, 5].

Based on a number of Next Generation Sequencing studies involving hundreds of melanomas, a molecular disease model of melanoma has been proposed, whereby ~40-50% of melanomas from patients carry mutations in the *BRAF* gene, with ~90% of these *BRAF* mutations being a *BRAF^V600E^* mutation, and a further 20% carry mutations in the *NRAS* gene [6]. Almost half a decade of Next Generation Sequencing studies of cutaneous melanoma has recently been reviewed [7], including one of the most recent and most extensive exome sequencing studies carried out to date. The Cancer Genome Atlas (TCGA) melanoma skin cancer study investigated exome sequences of 333 melanomas [8], and revealed recurrent aberrant sequence variants in a number of genes, including *BRAF*, *NRAS*, *TP53*, *PPP6C*, *NF1*, *CDKN2A*, *PTEN*, *ARID2*, *DDX3X*, *RAC1*, *IDH1*, *RB1*, *MAP2K1*, *HRAS*, *KRAS*, *KIT*, and *CDK4*.

*BRAF* and *NRAS* mutations result in activation of the MEK-ERK signalling cascade [9]. This observation has led to extensive efforts to develop drugs targeting this pathway. Two such drugs, Vemurafenib and Dabrafenib are BRAF inhibitors that inhibit mutant BRAF proteins containing V600E [10, 11], and prevent the activation of the MAP kinase pathway, resulting in antitumor effects such as inhibition of cell proliferation and induction of apoptosis [12].

In recent years BRAF inhibitor drugs have led to significantly improved outcomes for melanoma patients [9]. In addition, immune checkpoint inhibitors have been shown to significantly improve melanoma patient survival, and it is notable that better response rates to immune checkpoint inhibitors have been reported in melanomas with high mutation loads, particularly those containing *NRAS* mutations [13]. As progressively more therapies tailored towards oncogenic mutations are under development, it is important to understand the incidence of commonly mutated genes within a given population, especially in regard to targeted therapy options that may be available. Although the incidence rates of recurrent driver mutations in melanoma have been widely reported in different populations in the world, it remains unknown how frequent these mutations are in NZ melanomas.

Here we have investigated the mutation frequencies in 20 genes represented on the Sequenom MelaCarta MassARRAY in NZ melanomas. We included melanomas from both North and South Islands, constituting the first comprehensive mutation analysis of clinical melanoma samples in NZ.

## RESULTS

### Patients and samples

Genomic DNAs were isolated from 529 metastatic melanoma samples, from a total of 529 patients. Samples were analyzed using the Sequenom MassARRAY MelaCarta panel of recurrently mutated melanoma genes, and also by direct Sanger sequencing of *BRAF* exon 15, which served to identify the full extent of *BRAF* exon 15 mutations, and to provide validation of the *BRAF* MelaCarta mutation data.

**Figure 1:**
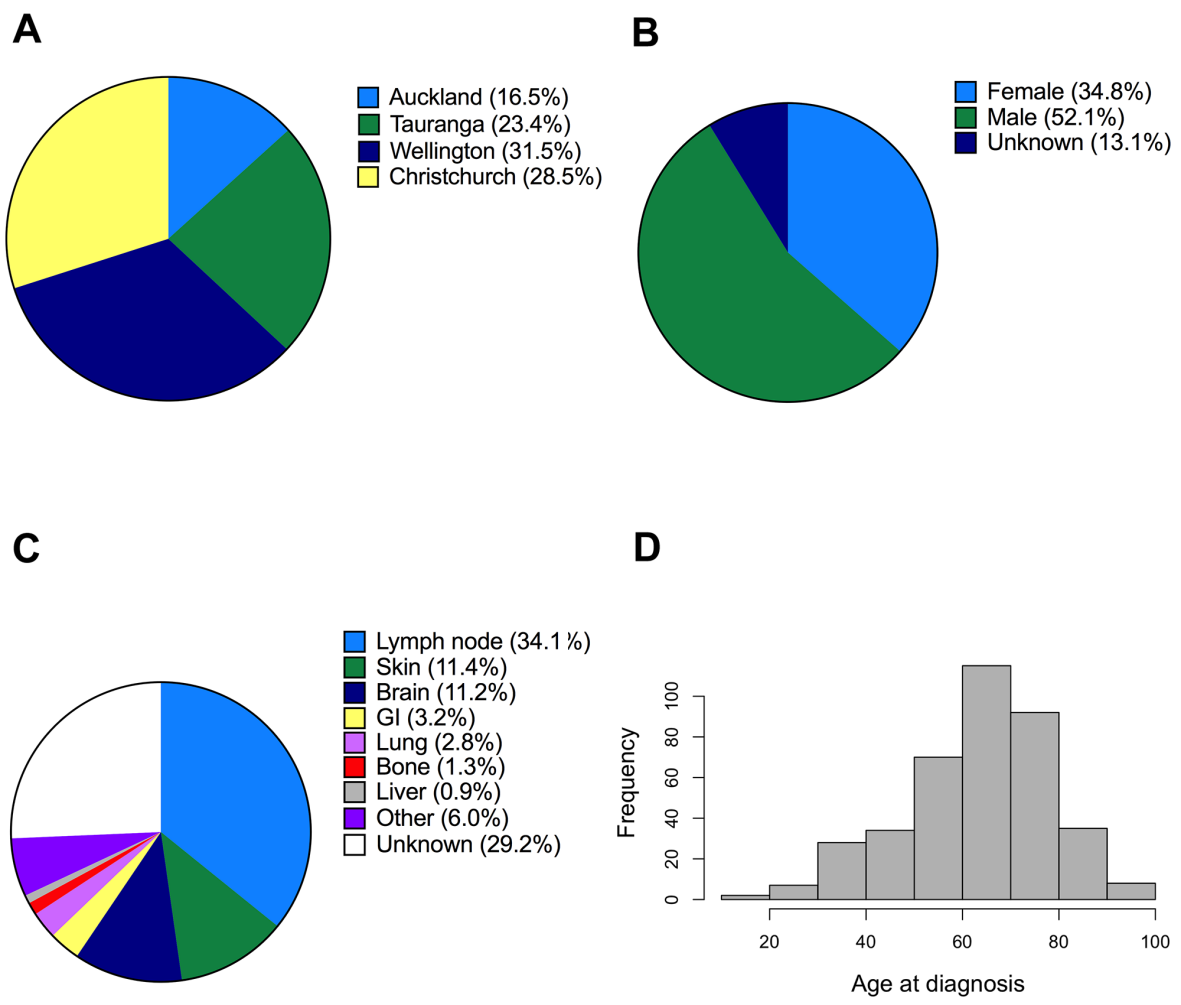
Description of metastatic melanoma patients analyzed in this study **A, B.** Geographical distribution and gender of the patients whose melanomas were analyzed for mutations using Sequenom MassARRAY (MelaCarta Panel v1.0). **C.** Distribution of the anatomical sites of the analyzed metastatic tumour samples. **D.** Histogram showing the age distribution of the patients.

Overall, 453 melanoma samples were successfully analyzed by Sanger sequencing, and 466 melanoma samples were successfully analyzed by Sequenom MelaCarta. Not all melanomas analyzed by sequencing were analyzed by MelaCarta and vice versa. The patients were diagnosed at four different geographically dispersed localities in NZ (Figure 1A), and were predominantly Caucasian (243 males, 162 females, 61 of unknown gender, Figure 1B). The most common location for metastasis in this cohort was lymph nodes (34.1% of the patients) followed by skin (11.4%) and brain (11.2%, Figure 1C). The age of the melanoma patients ranged from 18 to 95 years with the median being 66 years of age (Figure 1D).

### *BRAF* mutation analysis in New Zealand melanomas

Similar to previous studies [9], mutations in the *BRAF* oncogene were the most frequent in our cohort of NZ melanomas. For all melanomas analyzed (from all localities in NZ), *BRAF* mutations were identified in 33.1% of melanomas (175/529), and were detected by either MelaCarta and/or Sanger sequencing. Among *BRAF* mutations characterized by high resolution melting analysis (HRM) and Sanger sequencing we identified one novel *BRAF* mutation, *BRAF^L597H^* (Table 1). The majority of *BRAF* mutations were identified in both MelaCarta and Sanger sequencing platforms (Supplementary data Table S1), although 18/175 (10.3%) of all the *BRAF* mutations detected were only identified by Sanger sequencing, and were not detected by the MelaCarta platform. Of the exon 15 *BRAF* mutations not detected by MelaCarta, 11/18 (61.1%) were not included in the MelaCarta panel (Table 1). In contrast the MelaCarta panel detected an additional 4% of all *BRAF* mutations including *BRAF^G466A^* and *BRAF^G469E^* amino acid substitutions which lay outside of the exon 15 region sequenced by the Sanger method (Table 1).

**Table 1:** Predicted amino acid mutations in NZ melanomas identified in *BRAF* outside codon 600 detected using Sanger sequencing and/or Sequenom MelaCarta

*BRAF* Mutation	Geographical Location	Site of primary	Morphology of primary	Method of Detection	Present in MelaCarta
S607P	Wellington	Skin	NOS	Sanger	No
G606R	Tauranga	Trunk	NOS	Sanger	No
S605N	Auckland	Lower limb	NOS	Sanger	No
R603STOP	Christchurch	Lower limb	NOS	Sanger	No
K601E	Auckland	Upper limb	SSM	Sanger & Sequenom	Yes
K601E	Wellington	Trunk	nodular	Sanger & Sequenom	Yes
K601E	Christchurch	Upper limb	nodular	Sanger & Sequenom	Yes
K601N	Wellington	Scalp/Neck	nodular	Sanger	No
L597H**	Wellington	Upper limb	NOS	Sanger	No
L597S	Tauranga	Skin	NOS	Sanger	Yes
L597Q	Wellington	Spine	NOS	Sequenom	Yes
V600E and L597Q	Wellington	Trunk	SSM	Sanger	Yes
V600E and G596D	Tauranga	Trunk	NOS	Sanger	G596D is not in Melacarta
G596R	Auckland	unavailable	unavailable	Sanger	No
F595L	Auckland	Scalp/Neck	nodular	Sanger	No
D594A	Wellington	Lower limb	SSM	Sanger	No
D594E	Auckland	Ear	NOS	Sanger	No
D594N	Wellington	Scalp/Neck	nodular	Sanger	No
V590A*	Tauranga	Groin	NOS	Sanger	No
H585Y*	Wellington	Skin	amelanotic	Sanger	No
H585Y*	Wellington	Skin	NOS	Sanger	No
L584F	Wellington	Upper limb	NOS	Sanger	No
G469E	Auckland	unavaliable	NOS	Sequenom	Yes
G469E	Christchurch	Lymph Node	superficial spreading	Sequenom	Yes
G469E	Wellington	Urinary Bladder	NOS	Sequenom	Yes
G466A	Wellington	Small Bowel	nodular	Sequenom	Yes

Using Sanger sequencing we identified a previously unreported *BRAF* mutation at codon 597

** L597H c.1790_1791TA>AT

* two additional mutations that have not been associated with melanoma in earlier reports

Of the mutations identified in *BRAF, BRAF^V600E^* substitutions were the most common mutation type, comprising 73.7% of all *BRAF* mutations. The frequency of V600E mutations was 23.4% (109/466) of all melanomas identified by the MelaCarta panel alone or 24.4% (129/529) of all melanomas analyzed by MelaCarta plus Sanger sequencing. *BRAF^V600K^* mutations were detected in 4.3-4.5% of melanoma samples (MelaCarta, 21/466; or MelaCarta plus Sanger, 23/529) and these comprised 13.2%-15.0% of *BRAF* mutations detected. Patient age at the time of diagnosis of the first distant metastasis was available for 391 of the 466 patients (for MelaCarta assays). Patients with *BRAF* mutant metastatic melanoma were significantly younger at diagnosis of the first distant metastasis (mean = 59.1 years; SD=14.9) compared to the patients with *BRAF* wild-type melanoma (mean= 65.4 years; SD= 14.5; P= 0.0001, Student's *t*-test). Patients with *BRAF^V600E^* mutant metastatic melanoma were also associated with a significantly younger age (median= 57.0 years) compared to patients with *BRAF* wild-type melanoma (median= 67.0, P= 2.5 × 10^−7^, Mann-Whitney test). By comparison to *BRAF^V600E^* mutant metastatic melanomas, the *BRAF^V600K^* mutation was significantly associated with older age (median= 66.5) at diagnosis of the first distant metastasis (P= 0.0053, Mann-Whitney test). Similar associations with age and *BRAF* mutation status were previously reported [14, 15]. Patient gender was not associated with any *BRAF* mutant genotype.

**Figure 2:**
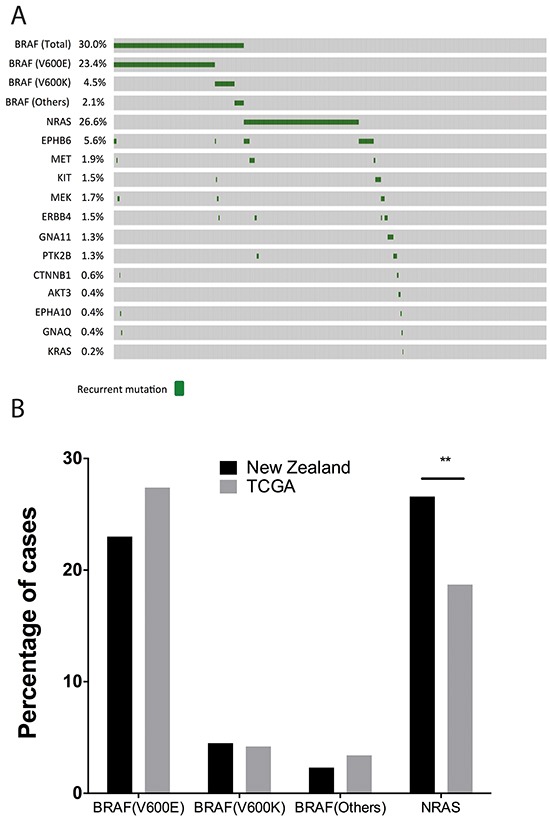
Overview of the mutational landscape in New Zealand population **A.** Oncoprint of mutations identified with the Sequenom MassARRAY (MelaCarta Panel) in 466 patients. The oncoprint was generated using cBioportal tools. **B.** Comparison of *BRAF* and *NRAS* mutations in the NZ population with the TCGA patient cohort.

The Cancer Genome Atlas (SKCM data in TCGA) (Figure 2B) currently includes mutation information for 368 metastatic melanoma patients derived from exome sequencing data. Comparing *BRAF* mutation rates in NZ melanomas with the melanoma mutation data from TCGA, we found that *BRAF^V600E^* mutations occurred in a relatively smaller percentage of NZ melanomas than in TCGA, although this was not significant (Figure 2B).

### Analysis of mutations in an additional 19 genes in the MelaCarta panel in New Zealand melanoma patients

The MelaCarta panel v1.0 [16] includes a further 59 somatic mutations, which are frequently altered in melanoma in a further 19 genes in addition to *BRAF*. We analyzed these mutations in 466 melanomas, consisting of 333 melanoma patients from the North Island of NZ, from Auckland, Tauranga and Wellington, and 133 melanoma patients from South Island, from Christchurch (see Figure 1A). Mutations in *NRAS* were identified in 26.6% of melanomas (124/466) (Figure 2). The *NRAS^Q61^* mutation was identified in 24.5% (115/466), and *NRAS^G12/13^* mutations were identified in 2.0% (9/466) of melanomas. *NRAS* mutations were significantly associated with older age (Table 2; median age of patients with *NRAS* mutation = 69; median age of patients with *BRAF* mutation = 60; median age of patients wild type for *BRAF* and *NRAS* mutations = 66, P = 0.0000274, Kruskal Wallis test), which is consistent with previously published data from USA, Australian and European populations [17–19]. Mutation status was also significantly associated with the site of the primary tumor (P= 0.004, Chi-square test, Table 2); *BRAF* and *NRAS* mutations were relatively more common in melanomas from the trunk (23.6% and 21.0%) compared to tumors without mutations detected (Wild Type) (15.3%). In contrast, *NRAS* mutations were more common in melanomas arising from the arm/leg extremities (45.2%) compared with *BRAF* mutant (30.0%) or melanomas without mutations detected (33.7%) for this location. Melanomas with no detectable *BRAF* or *NRAS* mutations were more common in head and neck melanomas (19.8%) compared to either *BRAF* (10.0%) or *NRAS* (8.9%) mutant melanomas. These associations are consistent with previous reports [17, 19].

**Table 2:** Clinical and pathological characteristics, and their association with four genotypes: *BRAF* mutation, *NRAS* mutation, *BRAF* and *NRAS* mutations not detected (Wild Type, WT), and *EPHB6* mutation

Clinical and pathological factors	All patients No.	*BRAF* No. (%)	*NRAS* No. (%)	WT No (%)	3 group P-value	*EPHB6* No. (%)
**No. of patients**	466	140 (30.0)	124 (26.6)	202 (43.3)		26
**Age at diagnosis (years)**						
Median	66	60	69	66	2.74 × 10^−5^	64
**Gender**						
Male	243	78 (55.7)	64 (51.6)	101 (50.0)	0.7564	9 (34.6)
Female	162	45 (32.1)	46 (37.1)	71 (35.1)		14 (53.8)
Unknown	61	17 (12.1)	14 (11.3)	30 (14.9)		3 (11.5)
**Primary tumor site**						
Trunk	90	33 (23.6)	26 (21.0)	31 (15.3)	0.004	5 (19.2)
Extremity	166	42 (30.0)	56 (45.2)	68 (33.7)		11 (42.3)
Head/Neck	65	14 (10.0)	11(8.9)	40 (19.8)		3 (11.5)
Unknown	123	51 (36.4)	31(25.0)	63 (31.2)		7 (26.9)
**Primary tumor histology**						
Superficial Spreading	103	33 (23.6)	26 (20.1)	44 (21.8)	0.034	6 (23.1)
Nodular	67	12 (8.6)	24 (19.4)	31 (15.3)		6 (23.1)
Acral Lentiginous	4	1 (0.7)	0	3 (1.5)		1 (4.3)
Lentigo Maligna	15	4 (2.9)	2 (1.6)	9 (4.5)		0
Spindle	5	0	1 (0.8)	4 (2.0)		0
Desmoplastic	3	0	0	3 (1.5)		0
NOS	196	70 (50.0)	54 (43.5)	72 (35.6)		11 (42.3)
Other*	10	3 (2.1)	5 (4.0)	2 (1.0)		1 (4.3)
Unknown	41	17 (12.1)	12 (9.7)	34 (16.8)		1 (4.3)
**No. of patients**	403	123	112	168		25
**Breslow thickness (mm)**						
**≤2**	265	72 (58.5)	75 (67.0)	118 (70.2)	0.1183	16 (64.0)
**2.1-4.0**	52	15 (12.2)	17 (15.2)	20 (11.9)		3 (12.0)
**>4**	86	36 (29.3)	20 (17.9)	30 (17.9)		6 (24.0)

* Other consists of blue naevus, and epithelioid cell melanoma.

The gene mutation status was also significantly associated with the histology of the primary tumor (P=0.034). Thirty-three tumors (23.6%) harboring *BRAF* mutations were identified as superficial spreading melanomas (SSM) compared with 26 (20.1%) melanomas with *NRAS* mutations (Table 2). In contrast, *NRAS* mutations were more commonly found in nodular melanomas (19.4%) compared to either *BRAF* mutant (8.6%) or WT tumors (15.3%). No statistically significant mutational associations with Breslow thickness were observed, although a slightly higher percentage of *BRAF* mutations occurred in melanomas >4mm thick. These findings are consistent with reports from previous studies [17].

### Melanomas from South Island patients have a significantly higher incidence of *NRAS* mutations, while melanomas from North Island patients have a significantly higher incidence of *EPHB6^G404S^* mutations

**Figure 3:**
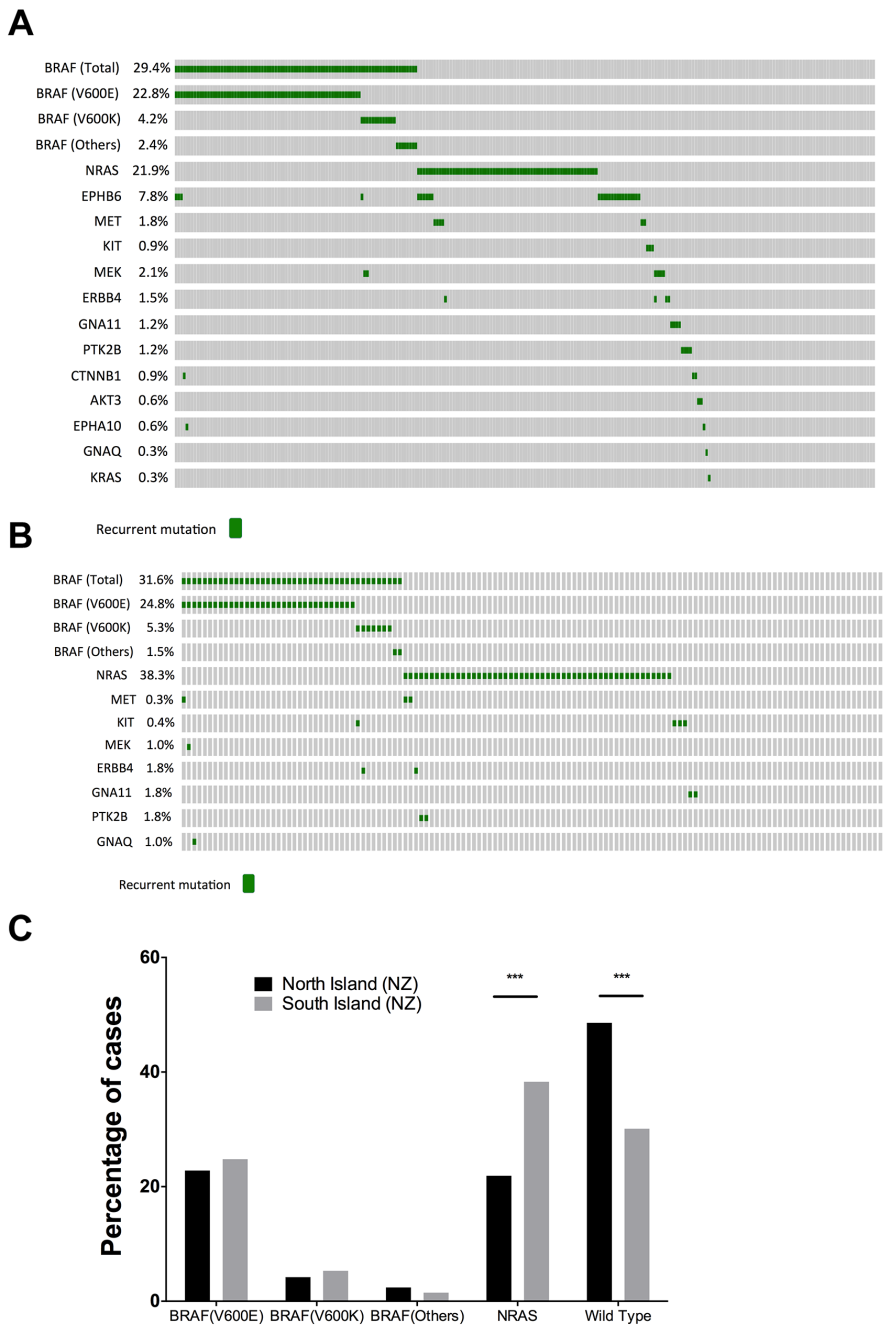
Comparison of *BRAF* and *NRAS* mutation profiles between North and South island melanomas Oncoprints of mutations in **A.** North and **B.** South Island melanomas. **C.** Comparison of the mutation frequencies of *BRAF* and *NRAS* mutations in North and South Islands. South Island melanomas had a significantly higher prevalence of *NRAS* mutant melanomas (P= 0.0004, Fishers exact test).

Melanomas from patients in the North and South Islands of NZ were analyzed to assess mutation incidence in association with different geographic locations (Figure 3A, 3B). We found that while the *BRAF* mutation (*BRAF^V600E/K^*) frequencies between the North and South Island were similar (Figure 3C), the prevalence of *NRAS* mutations in the South Island cohort (38.3%; 51/133) was significantly higher than in North island patients (21.9%; 73/333, P= 0.0005, Figure 3C). In addition, the *NRAS* mutation frequency in South Island was significantly elevated when compared to the *NRAS* mutation rate in TCGA data (22.0%; 81/368, P= 0. 0004). In contrast, the North Island *NRAS* mutation frequency was not significantly different from TCGA (21.9% versus 22.0%). Using logistical modeling, the expected odds ratio of *NRAS* mutations in melanomas in the South Island was 2.35 times that of the North Island with a 95% confidence interval ([1.50, 3.70], P=0.00016.)

In contrast, 5.6% (26/466) of melanoma samples contained an *EPHB6^G404S^* mutation, and when divided between North and South Islands, 7.8% (26/333) of North Island melanoma samples contained an *EPHB6^G404S^* mutation, making this the third most commonly mutated protein-coding gene after *BRAF* and *NRAS*. Remarkably, all 26 *EPHB6*^G404S^ mutation were observed in North Island melanomas, and *EPHB6^G404S^* mutations were not identified in South Island melanomas (P=0.0002). Closer inspection of MelaCarta data revealed that two *EPHB6^G404S^* mutations were present at very low levels in South Island melanomas, but were not identified by the MelaCarta software. Melanomas containing *EPHB6^G404S^* mutations in the North Island were identified from the following centres; Auckland n=6, Tauranga n=9, Wellington n=11. Overall, *EPHB6^G404S^* mutations were more frequently observed in melanomas without *BRAF* or *NRAS* mutations (16/202 melanomas, 7.9%), than co-occurring with either *BRAF* or *NRAS* mutations (10/264 melanomas, 3.8%, p-value = 0.04).

In addition to the *BRAF*, *NRAS* and *EPHB6* mutations, a small proportion of samples (<2% each, but in total comprising ~11.2% of patients) harbored mutations in *MET*, *KIT*, *MEK*, *ERBB4*, *GNA11*, *PTK2B*, *CTNNB1*, *AKT3*, *EPHA10*, *GNAQ* and *KRAS* genes (Figures 2A, 3A, 3B). Furthermore, 33/529 melanomas (6.2%) carried mutations in two or more genes in the MelaCarta panel. The co-detected additional mutations identified using the Melacarta Sequenom panel included six melanomas containing *NRAS* together with *EPHB6^G404S^* mutation, and six melanomas containing *NRAS* together with *MET^T992I^* mutation (Table 3).

**Table 3:** Additional mutations identified by Sequenom Melacarta analysis in NZ melanomas

**First mutation:**	*BRAF^V600E/K^*	*NRAS^Q61K/R/H/L^*	*ERBB4^E452K^*
**Second mutation:**	*EPHB6^G404S^* (4)	*EPHB6^G404S^* (6)	*MEK^P124L^* (1)
	*MEK^P124L^* (4)	*MET^T992I^* (6)	
	*MET^T992I^* (1)	*ERBB4^E452K^* (2)	
	*EPHA10^E124K^* (1)	*PTK2B^R429C^* (1)	
	*GNAQ^R183Q^* (1)	*PTK2B^G414V^* (1)	
	*CTNNB1^S45F^* (1)		
	*ERBB4^E452K^* (1)		
	*KIT^V559A^* (1)		

The amino acid substitutions (such as *BRAF^V600E^* or *BRAF^V600K^*) shown in the first line of the table indicate the initial mutation identified by the Melacarta analysis. The additional mutation is shown in the lines below the respective initial mutation. The number in brackets indicates the number of times the respective mutation combination was observed.

## DISCUSSION

Here we report the frequencies of recurrent mutations in NZ melanomas, including mutations in *BRAF*, *NRAS*, and *EPHB6*. Recurrent mutations in *BRAF* and *NRAS* in melanoma constitutively activate the mitogen-activated protein kinase (MAPK) signal transduction pathway, and *NRAS* mutations can also strongly activate the PI3/AKT pathway [20], leading to the activation of multiple downstream signaling pathways to promote cell proliferation, growth and survival. Although the frequencies of gene mutations have been reported in several international studies, mutation frequencies in NZ melanomas have not been reported to date.

We analyzed a large series of melanomas (n = 529) from different locations in NZ. Overall, *BRAF* mutations were observed in approximately one third of melanomas in NZ (175/529; 33.1%). *BRAF* mutations are usually detected in approximately 40-50% of melanomas [21]. Mutations in *BRAF^V600E^* (comprising 73.7% of *BRAF* mutations), and *BRAF^V600K^* (comprising 15.0% of *BRAF* mutations) made up most of the *BRAF* mutations. Sequenom analysis detected *BRAF* mutations (25.3%) at almost the same rate as Sanger Sequencing (25.7%) within codon 600. The concordance of *BRAF^V600^* mutation detection between the two methods was 91.9% (Supplementary data Table S1). Our data suggest that 22.1% of *BRAF* mutations in NZ melanomas were non-V600E mutations, which is similar to the 25% figure obtained in a study of 1,112 cases of melanoma using pyrosequencing or Sequenom MassARRAY [22]. Sanger sequencing detected more mutations at other locations in exon 15 of *BRAF* than was detected using Sequenom MassARRAY, an observation that is highly relevant to mutation testing for clinical diagnosis. In addition, the *BRAF* COBAS test (Roche Diagnostics) was carried out on ~200 of the patients analyzed in this study, and the concordance with Sanger sequencing was >95% (data not shown). While the *BRAF^V600E^* mutation rate was lower than that reported in previous mutation studies in melanoma [5, 23], *BRAF^V600K^* mutations were associated with chronic UV exposure and are more prevalent in geographical areas with the highest levels of UV radiation, including Australia and Texas where *BRAF^V600K^* mutations have been reported in 19% to 22.5% of total *BRAF* mutations, respectively [15, 19]. However we did not find an elevated incidence of the *BRAF^V600K^* mutation in NZ.

In a previous study from the USA, one of seventy-nine melanomas analyzed was reported to contain an *EPHB6^G404S^* mutation [24]. Mutations in *EPHB6* have been observed recurrently in other cancer types such as non-small cell lung cancer [25], but to our knowledge our study is the first to identify recurrent *EPHB6^G404S^* mutations in melanoma. The *EPHB6^G404S^* mutation involves substitution of glycine with serine in the Fibronectin Type III (FN3) domain of the EPHB membrane-bound protein tyrosine kinase, although the functional consequence of this mutation may be negligible, as structure/function analysis programs predicted it is benign [25]. Although we cannot rule out the possibility that the *EPHB6^G404^*sequence variant could be a germline polymorphism, this variant was not identified using Melacarta software in South Island patients, and it has not been previously reported as a polymorphic variant in four SNP databases (dbSNP/Uniprot, SNPper, ALFRED, or SNPedia). Interestingly, the *EPHB6^G404S^* mutation occurred significantly more frequently in melanomas without detectable *BRAF* or *NRAS* mutations.

Overall, *NRAS* mutations were detected in slightly less than a third (26.6%) of melanomas, and we found that *NRAS* mutations were much more frequent in melanomas of South Island patients (38.3%) than in North Island melanoma patients (21.9%), or in TCGA data (22%). The higher *NRAS* mutation frequency in South Island versus North Island was largely attributed to mutations occurring in codon 61 of *NRAS* exon 2. Therefore, *NRAS* exon 2 and *BRAF* exon 15 variants represented the two most frequently mutated exons in NZ melanomas. Further, in 6.2% of cases, the Melacarta Sequenom panel also identified a second mutation in addition to *BRAF* or *NRAS* mutations, with *NRAS* plus *EPHB6^G404S^* being one of the most frequent (Table 3).

Differences in *NRAS* mutation rates in other population groups have been reported previously. For example, markedly different *NRAS* mutation rates were reported between Middle-South Italy and Sardinia (21% vs. 2%; P<0.0001), and were suggested to be due to differences in genetic background [26], but no conclusive supporting evidence was presented for how the *NRAS* mutation rate became repressed in Sardinia.

Approximately 74% of the NZ population classify themselves as being of European decent, while only 14.9% and 11.8% of the population classify themselves as being Maori or Asian ethnicity respectively (http://www.stats.govt.nz/Census/2013-census/profile-and-summary-reports/quickstats-culture-identity/ethnic-groups-NZ.aspx). The proportion of Maori or Asian individuals in the NZ population influences melanoma incidence statistics in NZ, because individuals who are of Maori or Asian decent have markedly lower rates of melanoma than those of European decent in NZ (Wellington: Ministry of Health. 2015. www.health.govt.nz/publication/cancer-new-registrations-and-deaths-2012). Although almost 90% of people of Maori or Asian ethnicity in NZ reside in the North Island (www.stats.govt.nz/census), ethnicity was not considered a major contributory factor to differences in oncogene mutation rates observed in this study. The proportion of individuals who were of Maori or Asian decent was relatively small, and only very small differences in the numbers of Maori or Asian patients occurred between North and South Islands.

The main epidemiological risk factor contributing to NZ's high incidence of melanoma is annual UV radiation exposure. NZ has a 40% higher average UV exposure than North America [27] or central Europe [28]. Moreover, as discussed above, NZ has a mainly fair-skinned Caucasian population. However, the difference in the *NRAS* mutation frequency between North and South Islands may not directly be associated with DNA damage caused by UVB-radiation [4, 5, 23, 29]. This is because, while several recurrent oncogenic mutations in melanoma have been associated with UVB-radiation [3, 30], *BRAF^V600E^* and *NRAS^Q61R^* mutations do not themselves contain UVB-radiation mutation signatures. The conclusion that direct chronic UVB-induced DNA damage is not a key feature in NZ melanomas is also supported by the observation that *BRAF^V600K^* mutations were relatively infrequent in both North and South Island NZ melanoma cohorts.

UV radiation levels during summer in the South Island are on average lower than in the North Island, or in Queensland, Australia (~12°S to 27°S), but are nevertheless higher than similar latitudes in the Northern hemisphere [27]. Clear skies and a high UV index in South Island summers frequently lead to sunburn-associated erythema in fair skinned individuals. However, in winter UV radiation levels are relatively low in the South Island, and also in the Australian southern states, as compared to the rest of Australia, or to the North Island of NZ [27]. A relatively higher incidence of vitamin D deficiency occurs in Southern latitudes than the Northern latitudes of Australia and NZ [31–33], which raises the question of whether high UV-radiation exposure in South Island during Spring, when vitamin D levels are lowest [33], could lead to a high frequency of *NRAS^Q61^* mutations versus other mutation types. DNA mutations accumulate for up to 12 hours following acute sunburn, even in the dark, and vitamin D has been suggested to protect DNA from UV damage [34, 35]. Moreover, vitamin D deficiency at diagnosis is linked to higher Breslow thickness in melanoma [36]. In this study we found that *NRAS* mutations were associated with nodular melanomas, which on average had a higher Breslow thickness than other melanoma types. Regarding relative “skin age” in the South Island versus North Island, living in the South Island has the same effect as increasing skin age by 27 years.

Loss of a sun tan, lower vitamin D levels, and susceptibility to sunburns, are common following winter in the South Island of NZ, due to extreme seasonal difference in UV radiation [33]. In contrast, in many climates outdoor workers exposed to the sun all year do not have markedly increased melanoma rates [3]. If low levels of vitamin D potentially lead to higher *NRAS* mutation rates, this would involve a mechanism as yet not understood. *NRAS* mutations have previously been associated with UV-associated DNA damage [37–39], but are not frequently observed in melanomas containing high UV-induced mutation loads [40]. Although there is little or no available evidence to suggest that UVA-radiation can induce *NRAS* mutations in association with pheomelanin-induced reactive oxygen species (ROS) [41], absence of a UVB mutation signature in *NRAS* does not exclude this possibility.

High and low *NRAS* and *BRAF* mutation frequencies, respectively, have implications for the diagnosis, and treatment of melanoma in NZ. A lower rate of *BRAF^V600E^* mutations in NZ suggests that other *BRAF* mutation types may constitute a relatively higher proportion of the total *BRAF* mutations, and that mutation screening and treatment options should therefore take this into consideration. Also, the higher *NRAS* mutation rates in the South Island suggest that South Island melanoma patients could benefit more often from the use of immune checkpoint blockade therapy, which has higher response rates in *NRAS* mutant melanomas [13].

In conclusion, we show for the first time, in one of the largest cohorts of melanoma patients reported, that *NRAS* and *EPHB6* mutation frequencies in NZ melanomas are significantly variable between populations comprised of individuals of the same ethnic group, who have similar genetic backgrounds, and who have similar lifestyle practices and choices. We propose the differences in oncogene mutation frequencies observed in melanomas depends primarily on environmental risk factors, which we speculate could be associated with differences in intermittent exposure, or type of UV radiation of North and South Island NZ individuals. Understanding the reasons for variable mutation frequency will lead to precise interventions to effectively target oncogenic mutations, such as in *NRAS* or *EPHB6* in melanoma.

## MATERIALS AND METHODS

### Approval by ethics committee

Ethical approval for this study was obtained from the NZ Health and Disability Ethics Committee (HDEC reference numbers 13/CEN/46 and NZ/13/B7B408).

### Patient sample and clinical information collection

529 Formalin Fixed Paraffin Embedded (FFPE) samples of melanoma tissue from 529 patients and related clinical information were collected in the period 2000 to 2014 from five different laboratories in NZ, in (North Island) Auckland, Tauranga, Wellington, and (South Island) Christchurch. A total of 466 metastatic melanoma samples (one sample per patient) yielded Sequenom MassARRAY MelaCarta results, and 467 metastatic melanoma samples yielded Sanger sequencing results. Of these, 333 metastatic melanoma samples were from North Island, and 133 samples were from the South Island. Of the 333 samples from the North Island, 55 samples were from Auckland, 78 samples were from Tauranga and 105 samples were from Wellington. Patient clinical information was retrieved from the NZ Cancer Registry.

### DNA preparation

FFPE tissues were cut into 10 μm sections and mounted onto glass slides. Experienced pathologists histologically evaluated and determined areas with a large proportion of tumor cells on corresponding H&E slide for all melanoma samples. Tissues were macrodissected manually using a sterile blade in accordance with the marked areas of tissue on the H&E slides. Genomic DNAs were isolated using either a QIAmp DNA FFPE Tissue Kit or DNeasy kit (Qiagen) according to manufacturers instructions. The quantity and quality of DNA was assessed using a Nanodrop spectrophotometer and a Quantus Fluorometer (Promega).

### Gene mutations analyses

#### MelaCarta mass spectrometric analysis

466 of 529 melanoma samples yielded results for 72 somatic mutations in 20 melanoma-related genes using mass spectrometry genotyping on the Sequenom MassARRAY MelaCarta Panel v1.0 (Agena Bioscience, San Diego, CA, USA) and all assays were carried out by the Liggins Institute, University of Auckland, New Zealand. Mutation detection using an optimized mass spectrometric genotyping platform has been suggested to be more sensitive, but not necessarily more specific, than Sanger sequencing [42], although we also used high resolution melting analysis, which greatly improves the sensitivity of Sanger sequencing.

#### High resolution melting analysis

Approximately 85% of the samples analyzed by Sanger sequencing were also analyzed high resolution melting (HRM) analysis. HRM analysis was set up using 10 ng of genomic DNA and performed in duplicate on the LightCycler 480 (Roche) using previously published primers and amplification conditions [43]. Each run included a wildtype control, a homozygous mutant (*BRAF^V600E^*) and a low mutant control (40% mutant 60% wild-type) for normalization. The HRM results were analyzed by Gene Scanning software with normalized, temperature-shifted melting curves displayed as difference plots. Samples were considered mutated when significant difference of fluorescence level for all duplicates fell outside of the range of variation detected for the wild-type control.

#### Sanger sequencing of *BRAF* exon 15

Sanger sequencing remains the gold standard for mutation detection in clinical samples [44]. Bi-directional Sanger sequencing of all melanoma samples was carried out using the following protocol at the Capital and Coast District Health Board diagnostic laboratory, Wellington, New Zealand, to detect mutations in exon 15 of the *BRAF* gene with the following primers

BRAF 15-forward, 5′- TCATAATGCTTGCTCTGA TAGGA

BRAF 15-reverse, 3′-GGCCAAAAATTTAATCAG TGGA.

PCR was performed in a 25μl volume containing 50ng of genomic DNA, 0.5μl of polymerase (5U/μl Bioline), 2.5μl of reaction buffer (10x containing 1.5mmol/L MgCl2 (Bioline) 0.25μl of dNTPs (25μmol/L stock solution) and 0.5μl of each primer (20pmol each). PCR amplification was carried out under the following conditions: 95°C for 15 minutes followed by 40 cycles at 95°C for 30 seconds, 56°C for 30 seconds, 72°C for 30 seconds and a final extension step at 72°C for 10 minutes. Amplified products were purified using ExoSAP-IT (Affymetrix) and sequenced using the BigDye Terminator v3.1 Cycle Sequencing Kit (Applied Biosystems), according to the manufacturer's protocol. Sequences were run on an eight capillary ABI3500 genetic sequencer (Life Technologies).

### Data and statistical analyses

MelaCarta Panel outputs were processed using an excel spreadsheet. The TCGA mutation data was downloaded using the cBioportal tool on 8th August 2015. The downloaded data was level 3 and contained information (in text format) about the mutation status of each patient. From this information we segregated primary and metastatic melanomas and compared only metastatic melanoma mutations, as described.

### EU cohort

Fishers exact test was used to evaluate the association between mutational prevalence and between different populations. Statistical analysis was performed using the R studio (version 3.2.2) statistical software.

## SUPPLEMENTARY TABLE




