# Supplementary material for: NRAS and EPHB6 mutation rates differ in metastatic melanomas of patients in the North Island versus South Island of New Zealand
Source: Oncotarget. 2016 May 13;7(27):41017–30. doi: 10.18632/oncotarget.9351 (PMC5173039; doi:10.18632/oncotarget.9351)
Supplement: Supplementary file 1 [file oncotarget-07-41017-s001.pdf]

NRAS and *EPHB6* mutation rates differ in metastatic melanomas of patients in the North Island versus South Island of New Zealand

SUPPLEMENTARY TABLE

Supplementary Table S1: Concordance of mutation detection by Sanger Sequencing and Sequenom MelaCarta MassARRAY platforms

See Supplementary File 1
