# Supplementary material for: NRAS and EPHB6 mutation rates differ in metastatic melanomas of patients in the North Island versus South Island of New Zealand
Source: Oncotarget. 2016 May 13;7(27):41017–30. doi: 10.18632/oncotarget.9351 (PMC5173039; doi:10.18632/oncotarget.9351)
Supplement: Supplementary file 2 [file oncotarget-07-41017-s002.docx]

**SUPPLEMENTARY DATA**

| Combined Sanger & Sequenom; all *BRAF* mutations detected | Combined Sanger & Sequenom; *BRAF*^V600^ mutations detected | *BRAF^V600^* mutations detected by Sanger | *BRAF^V600^* mutations detected by MelaCarta | Concordant detection of *BRAF^V60^*^0^ mutations which were successfully analysed by both Sanger and MelaCarta | Combined Sanger & MelaCarta; non-*BRAF*^V600^ mutations detected | non-*BRAF^V600^* mutations detected by Sanger | non-*BRAF^V600^* mutations detected by MelaCarta | Concordant detection of non-*BRAF^V60^*^0^ mutations by both Sanger and MelaCarta |
| --- | --- | --- | --- | --- | --- | --- | --- | --- |
| 175/529 (33.1%) | 149/529 (28.1%) | 136/529 (25.7%) | 134/529 (25.3%) | 114/124 (91.9%) | 26/529 (4.9%) | 21/529 (4.0%) | 8/529 (1.5%) | 3/8 (37.5%) |

**Supplementary Table S1:** Concordance of mutation detection by Sanger Sequencing and Sequenom MelaCarta MassARRAY platforms.
